# Understanding when mixture models effectively identify careless responding: Simulation and validity evidence

**DOI:** 10.3758/s13428-026-03165-z

**Published:** 2026-10-09

**Authors:** Irina Uglanova, Gabriel Nagy, Esther Ulitzsch

**Affiliations:** 1 Leibniz Institute for Science and Mathematics Education (IPN), Olshausenstraße 62, 24118 Kiel, Germany; 2 Centre for Educational Measurement, University of Oslo, Oslo, Norway; 3 Centre for Research On Equality in Education, University of Oslo, Oslo, Norway

**Keywords:** Careless responding, Mixture modeling, IRT, Likert scale, Separability

## Abstract

Careless and insufficient effort responding (C/IER) in self-report questionnaires refers to responses provided without adequate attention to item content. Latent variable mixture models have recently been proposed as a promising approach for detecting and accounting for C/IER. This study systematically evaluates the performance of mixture modeling for C/IER detection. Using an exemplar mixture model that captures core features of existing approaches, we (a) investigated, via simulations, conditions that facilitate or impede separation between response patterns indicative of attentive versus careless responding and (b) examined the validity of interpreting the latent class variable as an indicator of C/IER in an empirical application. The simulation study assessed how questionnaire and item characteristics affect latent-class identification under different C/IER response patterns. Results indicate that reliable separation is achieved when scales comprise at least ten items, exhibit substantial threshold heterogeneity, and include both positively and negatively worded items. Empirical support for the C/IER interpretation of the latent class variable was obtained by reanalyzing a publicly available Big Five inventory data set collected across multiple platforms. Model-based classifications closely corresponded to alternative C/IER indicators that do not rely on response patterns, exhibited consistency across multiple scales, and were sensitive to platform-specific differences in C/IER prevalence. Overall, the results support mixture modeling as a viable approach for addressing C/IER under many conditions commonly encountered in applied research.

**Supplementary Information:**

The online version contains supplementary material available at https://doi.org/10.3758/s13428-026-03165-z.

## Introduction

Researchers who rely on self-report data face the challenge that some respondents do not read items carefully or do not invest sufficient effort when completing questionnaires. This behavior, referred to as careless and/or insufficient effort responding (C/IER; Curran, 2016), varies as a function of survey characteristics, respondent attributes such as personality (Bowling et al., 2016), and the broader assessment context (Ward & Meade, 2023), including the data-collection platform (Douglas et al., 2023; Kay, 2025; Peer et al., 2022; Suri et al., 2025). Because C/IER reduces data quality and threatens the validity of conclusions (Curran, 2016; Meade & Craig, 2012; Stosić et al., 2024; Ward & Meade, 2023; Welz et al., 2024), researchers need to evaluate the extent to which their data is affected. Common strategies are based on C/IER indices that summarize individual response patterns (Curran, 2016). More recent approaches rely on mixture models for response patterns (e.g., Arias et al., 2020; Jin et al., 2018), which avoid subjective classification decisions that are required in the case of C/IER indices.

Despite their growing use, little is known about the conditions under which these mixture models can reliably distinguish attentive from careless respondents based on their response patterns. Some studies have pointed to scale length as a relevant factor, suggesting that longer scales facilitate class separation (Jin & Chiu, 2024; Jin et al., 2018; Kam & Cheung, 2023). Additionally, including both positively and negatively worded items has been argued to be necessary to distinguish attentive and careless response patterns (Arias et al., 2023; Ulitzsch et al., 2022a, 2022b). However, researchers have not systematically examined the joint influence of these and other questionnaire and item characteristics. The first objective of this article is therefore to investigate, in a large simulation study, how questionnaire and item characteristics affect classification accuracy.

Even when respondents can be separated clearly, differences between latent classes may not exclusively reflect C/IER. Although several studies support interpreting the latent class variable as an indicator of C/IER (e.g., Arias et al., 2023; Kam & Cheung, 2023; van Laar & Braeken, 2022; Ulitzsch et al., 2022a, 2022b), further validation is needed. The second objective of this article is therefore to provide additional validity evidence. Using a publicly available Big Five inventory dataset (Douglas et al., 2023), we relate model-based classifications to alternative indicators of C/IER that are not based on response patterns to evaluate the interpretation of the latent class, and we compare the associations of these approaches with assessment conditions known to influence the prevalence of C/IER.

To ensure our findings generalize to a broad class of mixture models, we use a specification that retains the core features shared by most existing approaches, most notably a dichotomous latent class variable that classifies respondents as attentive or careless. At the same time, the model in focus avoids several restrictive assumptions in many prior specifications, such as treating item responses as continuous despite their categorical nature or imposing strong constraints on response patterns under C/IER. These assumptions can impede detection of careless responding that standard distributional forms do not capture well. By relaxing such constraints, the evaluated model reduces dependence on debatable assumptions and increases the likelihood that our results extend to closely related mixture modeling approaches for C/IER (Arias et al., 2020; Arias et al., 2023; Kam & Cheung, 2023; Steinmann et al., 2022; van Laar & Braeken, 2022; Ulitzsch et al., 2022a, 2022b).

## Using item response patterns to identify C/IER

C/IER reduces data quality and can, but must not necessarily, bias substantive conclusions. For example, Stosić et al. (2024), drawing on several published psychological studies, found that a C/IER rate of roughly 14% inflated correlations by about .07, and Welz et al. (2024) showed in simulation that even 5% C/IER can yield sign reversals in estimated correlations. Arias et al. (2020) demonstrated that removing as little as 4–10% of careless responders in an empirical dataset improved model fit. Patton et al. (2019) found that even 10% of C/IER can lead to substantial bias in item discrimination parameter estimates. Ulitzsch et al., (2024a, 2024b), in contrast, reported that cross-country comparisons of mean levels across multiple scales remained unchanged after accounting for an average C/IER rate of 6%. Because the degree of contamination by C/IER is unknown in advance, researchers cannot assume their data and conclusions drawn from them are unaffected—thus, evaluating the potential impact of C/IER on the results obtained after accounting for the (presumed) occurrence of C/IER against their unadjusted counterparts is essential for ensuring trustworthy results (Ward & Meade, 2023).

Many approaches for identifying C/IER utilize data that is collected in addition to item responses, such as attention checks (Huang et al., 2015; Kim et al., 2018) or process data such as response times and mouse movements (Bowling et al., 2023; Pokropek et al., 2023; Ulitzsch et al., 2024a, 2024b). However, these methods are not always applicable. In this article, we focus on approaches that detect C/IER based on item response patterns to substantive items and, as such, are broadly applicable. In the following, we review the two most common pattern-based approaches, namely C/IER indices and mixture models, and outline their similarities and differences. As we will show, both approaches rely on the same type of information, but mixture models offer the advantage of avoiding subjective decisions.

### Indicator-based approaches

Indicator-based approaches aim to detect C/IER-specific response patterns (for overviews, see Curran, 2016; DeSimone et al., 2015; Huang et al., 2015; Meade & Craig, 2012). These approaches can be grouped into three categories. First, C/IER can be conceptualized as atypical response patterns that deviate from attentive patterns. Because most respondents are expected to be attentive (Curran, 2016), researchers can identify these patterns using Mahalanobis distance to detect multivariate outliers (Mahalanobis, 1936) or person-fit statistics that quantify alignment with a measurement model (Jones et al., 2023; Wind & Wang, 2023). Second, overly consistent patterns, such as straightlining (SL) or strong respondent-specific category preferences (RCP)—a tendency to overuse a particular response category—can be detected using the long-string index (Johnson, 2005) or the intraindividual response variability index (Edwards, 2019). Third, overly inconsistent patterns, such as random responding (RR), can be captured by the even–odd consistency index, which correlates subscales composed of even- and odd-numbered items (Jackson, 1976, as cited in Johnson, 2005). Additional indices based on psychometric antonyms and synonyms have also proven effective in detecting response consistency (Goldammer et al., 2020). Because each index is sensitive to only one type of careless response behavior, researchers generally recommend combining multiple indices in a multihurdle approach (Curran, 2016; Dupuis et al., 2019; Goldammer et al., 2020; Hong et al., 2020).

### Mixture modeling

Most mixture models for C/IER assume that respondents belong to one of two latent groups (but see Jin et al., 2018; Jin & Chiu, 2024): attentive and C/IER. In these approaches, separate response models for each class are specified. In the attentive class, responses are assumed to reflect the latent traits being measured, so standard measurement models link observed item responses to the latent constructs. In the C/IER class, responses are assumed not to reflect the traits of interest, and the measurement model is specified accordingly.

**Fig. 1:**
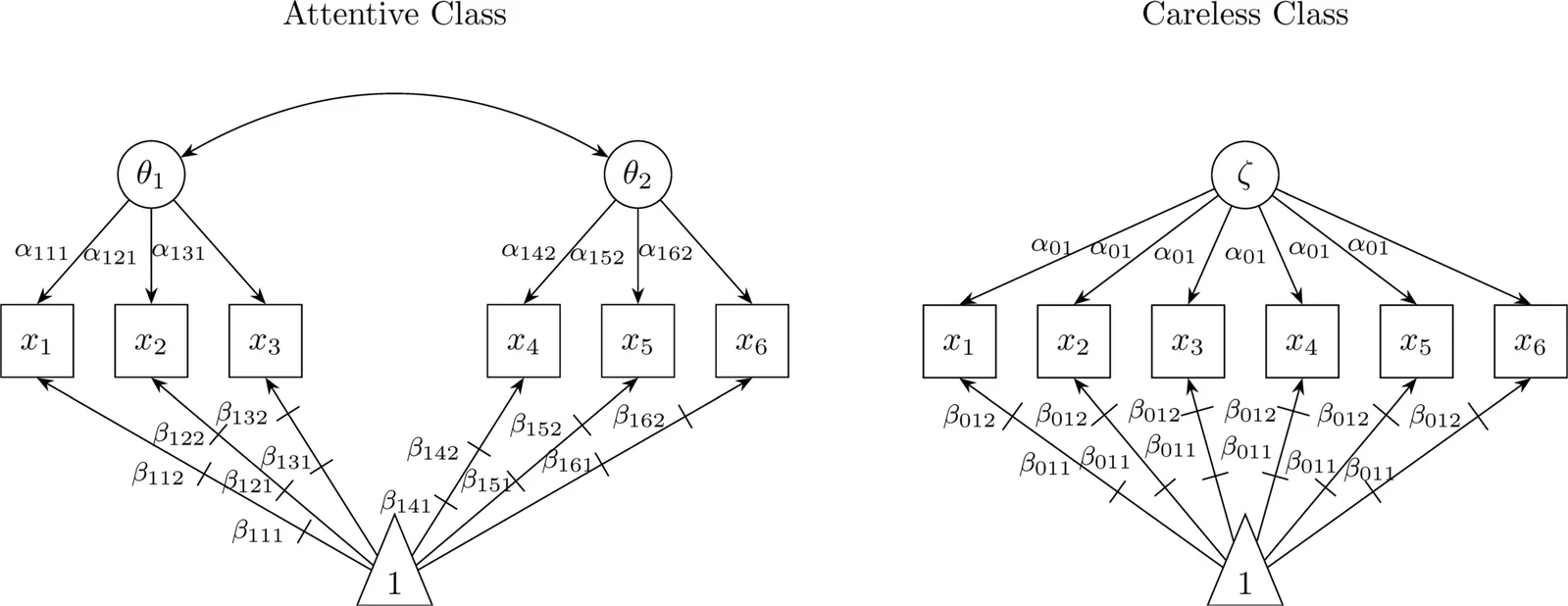
Path diagrams of a mixture model for C/IER. *Note*. ${\theta}_{1}$ and ${\theta}_{2}$ are the latent variables in the attentive class, ${\alpha}_{Cjt}$ are discrimination parameters, ${\beta}_{Cjk}$ are threshold parameters, ζ is the latent variable of the respondent’s category preference estimated in the careless class, ${x}_{1}\dots {x}_{6}$ are observed variables

Figure 1 presents a schematic path diagram that illustrates the key features of the attentive and the C/IER class. Note that the path diagram refers to an IRT-based measurement model that acknowledges the categorical nature of item responses. This feature is not included in all two-class mixture models for C/IER, as some treat items as continuous indicators in linear measurement models (e.g., Arias et al., 2020; Arias et al., 2023; Kam & Cheung, 2023). Treating items as categorical is more general because it allows for differences in the shape of the marginal response distributions between classes (e.g., normal in attentive respondents vs. uniform in C/IER), whereas linear factor models cannot handle such differences. In this example, in the attentive class (*C* = 1), a two-dimensional measurement model with a simple structure in which the latent traits ${\theta}_{1}$ and ${\theta}_{2}$ affect responses on item *j* (*j* = 1, 2, …, *J*) with *K* response categories (*k* = *1, …, K − 1* thresholds) is assumed. The α-parameters stand for item discriminations, whereas the β-parameters are response category thresholds. The α- and β-parameters are allowed to differ between items, meaning that items could be more or less indicative of the latent traits $({\alpha}_{1jt}$) and items could be easier or harder to endorse (${\beta}_{1jk}$).

In the C/IER class (*C* = 0), item properties are specified not to differ between items because responses are assumed not to depend on the item content. Hence, the β-parameters are set equal in this class (i.e., ${\beta}_{0jk}={\beta}_{0k}$ for all *j* = 1, 2, …, *J*). Nevertheless, even in the case of C/IER, responses could depend on the individuals’ preferences for specific response options (e.g., higher or lower response options). Therefore, the model depicted in Fig. 1 includes one common latent variable ζ that replaces the substantive traits. Because in the case of C/IER all items are assumed to equally reflect preferences for response options (ζ), the loadings are set equal across items (i.e., ${\alpha}_{0j1}={\alpha}_{01}$ for all *j* = 1, 2, …, *J*). Note that not all models used in practice allow for individual differences in response category preferences (Roman et al., 2024; Ulitzsch et al., 2022a, 2022b; van Laar & Braeken, 2022; Zhang et al., 2025a, 2025b).

Overall, the two-class model for C/IER depicted in Fig. 1 shares the key aspects of most models suggested in the literature (e.g., Arias et al., 2020; Arias et al., 2023; Kam & Cheung, 2023; Steinmann et al., 2022; van Laar & Braeken, 2022; Ulitzsch et al., 2022a, 2022b). However, the model is more general because it can accommodate dependencies between item responses in the C/IER class, and it accounts for differences in response distributions between attentive and C/IER responses.

### Relationships between C/IER indicators and mixture models

Indicator-based approaches and mixture models build on the same type of data and share the assumption that, in the case of C/IER, similar types of response patterns occur. For example, atypical response patterns flagged by Mahalanobis as C/IER could be expected to be likely to be assigned to the C/IER class of mixture models. Likewise, patterns identified as overly consistent or inconsistent by C/IER indicators such as the long-string, intraindividual response variability, or even–odd consistency indices are likely to be classified as C/IER in appropriately specified mixture models. Indeed, indicator-based approaches and mixture models often yielded converging results (e.g., Arias et al., 2020, 2023).

Indicator-based approaches and mixture models differ from each other in how they classify C/IER. Indicator-based approaches require researchers to decide which individuals should be classified as C/IER by specifying threshold values (hurdles) for the indicators used (e.g., Goldammer et al., 2020; Hong et al., 2020). To the best of our knowledge, no clearly theoretically or statistically grounded rules for setting thresholds exist, which means that classifications of C/IER provided by response-pattern-based indicators are sensitive to researcher-chosen threshold values, creating considerable researcher degrees of freedom. Previous research has repeatedly shown that minor differences in threshold-setting choices lead to substantially different classification rates (Niessen et al., 2016; Ulitzsch et al., 2022a, 2022b; Vogelsmeier et al., 2025).

In contrast, mixture models provide probability-based classifications (posterior class probabilities) that are derived on the basis of statistical models with transparent measurement and distributional assumptions. Model-based classifications can be said to be more effective in communicating the certainty with which individuals are classified as attentive or C/IER. Rather than reducing classification to a binary decision, posterior class probabilities convey the degree of certainty associated with each individual classification. For example, a posterior probability of.60 indicates that the observed response pattern provides little information about whether the respondent answered attentively or carelessly, based on the employed model and its assumptions about each type of behavior. In contrast, a posterior probability of.90, for example, implies much greater classification certainty, providing a clear indication of whether the individual’s responses do or do not contain information about their trait values, based on the chosen model. Therefore, some researchers have concluded that mixture models are superior to indicator-based multiple hurdle approaches (Arias et al., 2020, 2023).

## Evaluating model-based classifications of C/IER

Although some researchers expect mixture models to classify C/IER more effectively than indicator-based approaches (Arias et al., 2020, 2023), several practical challenges remain insufficiently understood. Two issues in particular call for a systematic empirical evaluation. First, mixture-model performance depends on the degree of latent-class separation (Depaoli, 2013; Raykov et al., 2020). This raises the question of which questionnaire characteristics are most likely to enhance class separability in applied settings—that is, how well response patterns expected under C/IER can be distinguished from those expected for attentive respondents. Second, even when a model yields clearly separated classes, these groups may still reflect processes other than C/IER. Therefore, empirical evidence is needed to support the validity of interpreting the latent class variable in terms of C/IER.

### Prerequisites for latent class separation in mixture models for C/IER

Class separation depends on how strongly response patterns differ between classes (Depaoli, 2013; Raykov et al., 2020), and can largely be determined by the characteristics of the scales at hand. For instance, it has been argued that longer scales (Edwards, 2019; Jin & Chiu, 2024; Jin et al., 2018; Kam & Cheung, 2023) and the use of positively and negatively worded items help to disentangle response patterns that are typical for attentive and careless responses (Arias et al., 2023; Dunn et al., 2018; Ulitzsch et al., 2022a, 2022b). Longer scales help to detect differences between patterns. For instance, with only three items, a response pattern that includes only one category (e.g., “3”, “3”, “3”) has a relatively high likelihood of occurring under attentive responding, SL, or even RR.

Scales composed of positively and negatively worded items can be expected to result in specifically structured response profiles under attentive responding that make it easier for the model to distinguish them from careless response patterns. If all items are worded in the same direction, an attentive response pattern is relatively likely to include only one response category. In contrast, with a mixed-worded scale, attentive respondents are expected to choose categories on opposite sides of the response scale for items worded in different directions, whereas respondents exhibiting RCP (or SL) are expected to select the same response category regardless of the direction of the items’ wording.

A similar consideration can be made for scales with items that cover a broad, as opposed to a narrow range of content (i.e., bandwidth–fidelity dilemma; Cronbach & Gleser, 1957; Salgado, 2017). For example, broad personality scales such as the NEO-PI-R (Costa & McCrae, 1992), which cover a wide range of behavioral content, represent favorable conditions for class separation, whereas scales measuring highly specific constructs or disorders, such as the GAD-7 (Löwe et al., 2008), whose items all refer to similar anxiety-related experiences, represent more challenging conditions. For attentive respondents, one may expect pronounced differences in marginal response distributions across such items. This is different for careless respondents who do not pay attention to item content and, as such, can be expected to produce responses that share the same marginal distribution across all items. This suggests that questionnaires mixing items with high and low endorsement probabilities, which implies a wider spread of thresholds across items (high item heterogeneity), lead to better separation.

These considerations illustrate how questionnaire design can affect class separation. However, real applications are more complex because separation is very likely to be influenced by additional features such as questionnaire length and the specific response patterns that arise under C/IER. A comprehensive evaluation of the conditions under which mixture models reliably identify latent classes, therefore, requires studying multiple factors, some under the control of researchers, such as item wording, and others that cannot be manipulated, such as the characteristics of responses in the case of C/IER (i.e., different C/IER types such as RR, RCP, and SL).

### Validity evidence for interpreting the latent classes in terms of C/IER

Even when latent classes are well separated, there is no guarantee that mixture modeling approaches primarily capture C/IER. Prior studies have gathered validity evidence from several sources to support the interpretation of the latent class variable as an indicator of C/IER. Validity evidence related to internal structure could be gathered by establishing improved model fit and/or psychometric properties upon accounting for C/IER with mixture modeling approaches (e.g., Arias et al., 2020, 2023; Cao et al., 2024; Zhang et al., 2025a, b). Further, multiple studies gathered validity evidence through establishing agreement with alternative measures of C/IER, such as pattern-based indicators (e.g., Jin et al., 2018; Kam & Cheung, 2023; Ulitzsch et al., 2022a, 2022b), attention check items (Kam & Cheung, 2023), i.e., items specially designed to verify that respondents are attentively reading statements and follow instructions (Huang et al., 2015; Kim et al., 2018), or self-reported respondent burden (Vogelsmeier et al., 2024).

In the present study, in addition to replicating results on agreement with attention check items, we complement these cumulative efforts to gather validity evidence by investigating (a) relationships with study characteristics that can be assumed to be associated with C/IER occurrence and (b) consistency in C/IER classification across scales. Specifically, we evaluate the relationship with data collection platforms as an additional source of validity evidence: Multiple studies suggest that different online data collection platforms are more or less likely to attract respondents who only complete questionnaires for compensation, which renders C/IER more probable (Douglas et al., 2023; Kay, 2025; Peer et al., 2022; Suri et al., 2025). Hence, strong relationships between the latent class variable and data collection platform can be interpreted as additional empirical validity evidence supporting the interpretation of the latent class variable as an indicator of C/IER. Our investigations of consistency in C/IER classification across scales are based on the consideration that C/IER may reflect enduring inter-individual differences that generalize across time and research contexts (Bowling et al., 2016) and, for that matter, across jointly administered scales. Hence, consistency in C/IER classification can be interpreted as additional empirical validity evidence supporting the interpretation of the latent class variable (Ulitzsch et al., 2024a, 2024b).

## The present study

The primary goals of this study are twofold. First, we aim to examine which conditions allow separating latent classes on the basis of their response patterns. Second, we seek new empirical evidence that supports the interpretation of the latent class variable as an indicator of C/IER. To this end, we utilized an exemplar mixture IRT model (Fig. 1). The model follows the core feature of most existing models: it includes dichotomous latent class variables and assumes that responses in the C/IER class do not depend on item characteristics. Nevertheless, the model used here is more general, as it accounts for the categorical nature of item responses and can therefore represent different marginal distribution forms within the classes. Similarly, the model can account for dependencies in the C/IER class due to individual differences in category preferences. These features ensure that our results are not affected by debatable assumptions. At the same time, because the utilized model is very similar to typical models known from the literature, the essence of the results derived here can be expected to hold for other variants of mixture models for C/IER.

To address the first goal, we conducted a comprehensive simulation study, manipulating factors related to the scales’ characteristics (e.g., number of items, the presence of negatively worded items), the items’ characteristics (item thresholds and discrimination parameters), as well as the prevailing response pattern under C/IER. Based on these findings, we identify questionnaire characteristics under which mixture models can provide accurate C/IER detection.

To address the second goal, we reanalyzed a publicly available Big Five data set collected across four online platforms (Douglas et al., 2023). First, we examined the correspondence between the model-based classifications and an alternative indicator of C/IER, namely, attention check items. In contrast to mixture models, attention checks do not rely on response patterns. Instead, they directly assess attention to the item content by requiring an unambiguous and easily verifiable response (Huang et al., 2015; Kim et al., 2018). Second, we compared C/IER classifications across multiple data collection platforms, which are known to differ systematically in the quality of the data they provide (Douglas et al., 2023; Kay, 2025; Peer et al., 2022; Suri et al., 2025).

## Simulation study

The aim of the simulation study was to systematically evaluate the exemplar mixture model’s ability to differentiate between latent classes across a broad range of conditions. We based our investigations on three evaluation criteria: (a) the accuracy of model comparisons to determine whether C/IER is present in the data; (b) the accuracy of estimated C/IER proportions; and (c) the accuracy of individual-level classification.

### Factors affecting the separability between attentive and C/IER patterns

In this section, we describe the factors manipulated in the simulation study, guided by the questionnaire characteristics affecting latent class separation outlined above. We hypothesized that the number of items (Jin & Chiu, 2024; Jin et al., 2018; Kam & Cheung, 2023), the inclusion of positively and negatively worded items (Arias et al., 2023; Ulitzsch et al., 2022a, 2022b), and the measurement parameters of the attentive measurement model (thresholds and discriminations) influence the model’s ability to separate response patterns expected for attentive responders from different kinds of patterns expected under C/IER (SL, RR, or RCP). First, the latent class separation is hindered by the use of only a small number of items, which implies little information about response patterns (Jin & Chiu, 2024; Jin et al., 2018; Kam & Cheung, 2023). Second, attentive response patterns are expected to be hardly distinguishable from overly consistent patterns, such as SL or RCP, if (a) all items are worded in the same direction, (b) the thresholds are very similar across items (low item heterogeneity, e.g., all items are easy to endorse), and (c) the discrimination parameters are strong. If unidirectional item wording is combined with low threshold variability (low item heterogeneity), attentive respondents can be expected to use the same response category that best describes them across all items, making their response pattern resemble overly consistent C/IER patterns. Under these conditions, paradoxically, higher discrimination parameters might further hinder the differentiation between response patterns. If attentive respondents consistently select a particular response category that best describes them, their response pattern will be very similar to an overly consistent C/IER pattern.

### Evaluated mixture model

Like most mixture models for careless responding, our exemplar mixture model assumes two latent classes: an attentive class and a C/IER class (Fig. 1). Let *C*_*i*_ refer to the latent class of respondent *i (i* = 1, *…, N)*. For the attentive class, *C*_*i*_ = 1, the latent variable ${\theta}_{it}$ reflects the individual *i*’s standing on trait *t (t* = 1, *…, T)*. For the careless class, *C*_*i*_ = 0, only one latent variable is assumed, representing a respondent’s category preference, ${\zeta}_{i}$. Different IRT models can be used to model the responses to polytomous items. In the present study, we used a (multidimensional) graded response model, utilizing a logit link (Muraki & Carlson, 1995).

#### Attentive class of the mixture model

For items with *K* response categories and, consequently, with *k* = *1, …, K − 1* thresholds, the probability that respondent *i* in *C* = 1 chooses a category equal to or above *k* for item *j (j* = 1, *…, J)* that measures trait *t* as

1 $$P\left({x}_{ij}\ge k|{C}_{i}=1\right)=\frac{1}{1+\mathrm{exp}\left[-\left({\sum}_{t=1}^{T}{\alpha}_{1jt}{\theta}_{it}+{\beta}_{1jk}\right)\right]},$$

where ${\alpha}_{1jt}$ stands for the discrimination parameter of the *t*th dimension measured by item *j*, and ${\beta}_{1jk}$ is the threshold *k* of item *j* in *C* = 1. Note that, in the case of within-item dimensionality, item *j* could have up to *T* nonzero loadings. The model is based on raw (i.e., not recoded) item responses. This means that ${\alpha}_{1jt}$ can be expected to be negative for negatively worded items. For identification purposes, the mean of the latent trait is fixed to 0, and the variance of the trait distribution is fixed to 1.

#### C/IER class of the mixture model

The C/IER class measurement model assumes responses to be governed by the careless respondent’s category preference, ${\zeta}_{i}$. This preference is assumed to underlie all items, regardless of their content. Accordingly, under *C* = 0, we assume a unidimensional measurement model, where discrimination parameters are equal across items. In order to identify the scale of ζ, we fixed all loadings to 1 (i.e., ${\alpha}_{0j1}=1$ for all j=1,2,...,J) and estimate the variance of ζ. This specification is statistically equivalent to the specification presented in Fig. 1. In addition to this constraint, the threshold parameters ${\beta}_{0jk}$ are constrained to be equal for all $j=\mathrm{1,2},...,J$ items. This constraint reflects the assumption that in case of C/IER responses do not depend on the items’ content.

Hence, the probability that respondent *i* with *C*_*i*_ = 0 chooses a category equal to or above *k* for item* j* is represented as

2 $$P\left({x}_{ij}\ge k|{C}_{i}=0\right)=\frac{1}{1+\mathrm{exp}\left[-\left({\zeta}_{i}+{\beta}_{0k}\right)\right]}$$

Note that the C/IER class is intended to function as a “catch-all” class that captures various forms of careless response patterns. In Appendix A, we provide further detail on how the model accounts for different manifestations of C/IER, with a focus on SL, RCP, and RR.

### Design of the simulation study

For the simulation study, we considered a varying number of five-point Likert scale items (5, 10, and 20) from a unidimensional scale in a sample of 500 examinees. We decided on a unidimensional structure because it is likely to represent the most challenging scenario for the mixture model to disentangle respondents, since multidimensional scales include more items in most cases and, in addition, attentive respondents can be expected to show systematic shifts in their responses across weakly or negatively correlated scales, providing additional diagnostic information that is absent in the unidimensional case.

In order to examine the mixture IRT model’s ability to identify the presence of C/IER, we created scenarios where C/IER was present and where it was not. For the former, the attentive model (Eq. 1) was the data-generating model for 90% of the participants (attentive class). The remaining 10% constituted the C/IER class (Eq. 2), which was represented by one of four C/IER-types of response patterns: SL, RCP, RR, or a blend of RCP and RR (RCP + RR). In the second condition, the GRM (Eq. 1) was the data-generating model for the whole sample. The proportion of C/IER was fixed at 10%, which represents a common rate of contamination across many research contexts. Reported rates vary from approximately 5–12% in offline studies (Gibson & Bowling, 2020; Kim & Oh, 2023) and 6–10% in large-scale assessments (Bulut & Bulut, 2022; Ulitzsch et al., 2024a, 2024b; van Laar & Braeken, 2022), to 10–16% in online student samples (Arndt et al., 2021; Meade & Craig, 2012). Notably higher prevalence rates for C/IER were observed primarily for survey platforms that recruit respondents through payment systems. Here estimated proportions range from 11–32% on Prolific (Arias et al., 2020; Douglas et al., 2023) to 55–80% on MTurk (Arndt et al., 2021; Douglas et al., 2023; Hauser et al., 2019; Kim & Oh, 2023; Wessling et al., 2017). Thus, 10% reflects a conservative yet realistic baseline for many applied settings. It is worth noting that a 10% proportion means an extreme class imbalance and is therefore a challenging scenario for mixture models (e.g., Scrucca, 2023; Tueller & Lubke, 2010).

In the generation of attentive responses, we varied the semantic polarity of the items (0% or 40% negatively worded items), the amount of between-item differences in thresholds (low vs. high item heterogeneity, and the strength of the item discriminations (weak vs. strong). Under the condition of weak discrimination parameters, all discriminations in the attentive measurement model were set to an absolute value of |0.5| (equal to a standardized factor loading of |0.282|); under the condition of strong discrimination parameters, all discriminations were set to an absolute value of |2.00| (equal to a standardized factor loading of |0.762|). We expect a complex pattern of effects of discrimination parameter strength on the detection of C/IER that depends on the prevailing C/IER type. If SL occurs on items with stronger discriminations all worded in the same direction, attentive respondents tend to select the same response category consistently. Stronger discrimination sharpens this tendency further, rendering attentive patterns increasingly indistinguishable from SL. Under RR, by contrast, we expect the effect of discrimination strength on separability to be reversed: weak discrimination reduces the predictability of attentive responses, which may then resemble RR patterns.

Using the parameterization provided in Eq. (1), we considered five sets of equally-spaced item thresholds for each item heterogeneity condition (summarized in Table 1). For the low item heterogeneity condition, values for the first threshold in each set of five items ranged from 3.5 to 1.5. The difference between the first and the last threshold was set to 5 logits for all items (${\beta}_{j4}$ – ${\beta}_{j1}$ = 5 for all *j* = 1, 2, …, *J*). In contrast, for high item heterogeneity, condition values for the first threshold in each set of five items ranged from 5.00 to 1.00. The difference between the first and the last threshold of a given item was 6 logits (${\beta}_{j4}$ – ${\beta}_{j1}$ = 6 for all *j* = 1, 2, …, *J*). Examples of item characteristic curves for attentive item responses under different simulation conditions are provided in Appendix B.

**Table 1:** Thresholds under low and high heterogeneity conditions

Item Heterogeneity	${\beta}_{j1}$	${\beta}_{j2}$	${\beta}_{j3}$	${\beta}_{j4}$
Low	3.50	1.83	0.17	– 1.50
	3.00	1.33	– 0.33	– 2.00
	2.50	0.83	– 0.83	– 2.50
	2.00	0.33	– 1.33	– 3.00
	1.50	– 0.17	– 1.83	– 3.50
High	5.00	3.00	1.00	– 1.00
	4.00	2.00	0.00	– 2.00
	3.00	1.00	– 1.00	– 3.00
	2.00	0.00	– 2.00	– 4.00
	1.00	– 1.00	– 3.00	– 5.00

Columns represent threshold parameters, and rows correspond to items

The data generation for the careless responses was as follows:

1. SL: The first response was a random draw of one response category (with equal category probabilities), and the following responses were set to be identical.
2. RCP: The observed responses were generated with Eq. (2) as the data-generating model. The discrimination parameters were fixed to 1; threshold parameters were set to 5.00, 3.00, 1.00, and – 1.00 (see ICC in Appendix A, Fig. A2) for all items.
3. RR: Responses were randomly drawn with equal category probabilities.
4. RCP + RR: 5% of the C/IER respondents were generated to exhibit RCP; the remaining 5% were RR.

As all factors were crossed, the simulation study included 240 conditions. For each condition, 200 data sets were generated.

### Data analysis

Each data set was analyzed by the mixture model described above and a standard GRM that assumes that all item responses are attentive. For the estimation of both models, we employed the R package “*mirt*” (v1.41; Chalmers, 2012) using the expectation–maximization (EM) algorithm. We used 25 random starting values for the mixture model’s estimation, and we selected the solution with the highest log-likelihood (LL) to ensure that we found the global optimum (e.g., Asparouhov & Muthén, 2019). Note that label switching is precluded in the present study by the confirmatory nature of the model specification: the equality constraints on response thresholds and discriminations across items in the C/IER class render class labeling unambiguous by definition.

Recall that, under conditions where C/IER resembles SL, the latent variance of the latent variable in the C/IER class is expected to be very large (see Fig. 1). Therefore, to facilitate the estimation process, the starting value for the latent variance of the C/IER class was set to a very (admittedly arbitrarily) high value of 1000 in conditions involving SL. While other large starting values, such as 500 or 2000, are expected to be similarly effective in promoting model convergence, the sensitivity of results to the choice of starting value, especially under challenging conditions, deserves additional systematic investigation in future studies.

To evaluate the accuracy of conclusions about whether or not C/IER is present in the data, we followed the typical approach of deciding between a simple model that does not account for C/IER (i.e., the GRM) and the mixture model. To this end, we utilized the Bayesian information criterion (BIC), with a smaller value indicating a better fit, and selected the model with the smallest BIC. For data that has not been contaminated by C/IER, the GRM should be chosen. In cases where the C/IER is present, the mixture model should be preferred.

Next, we examined the estimated proportion of careless respondents. We considered situations where relative bias exceeds 10% (Muthén & Muthén, 2002) as problematic (i.e., where the average estimated C/IER proportion across replications was below 9% or above 11%). To inspect the accuracy of the individual-level classification, we investigated the sensitivity and specificity of the C/IER classifications. Specificity indicates the probability of a respondent being assigned to the attentive class (based on the posterior probabilities using modal assignment) if they were simulated as attentive. Sensitivity indicates the probability of a respondent being assigned to the C/IER class if they were simulated as C/IER. As a general approach used in practice, values close to 1 indicate high classification accuracy, with values above.90 suggesting that the vast majority of simulated cases were correctly classified (e.g., Plante & Vance, 1994; Šimundić, 2009). All analyses were conducted using R, Version 4.3.3 (2024).

## Results

### Model comparison

**Fig. 2:**
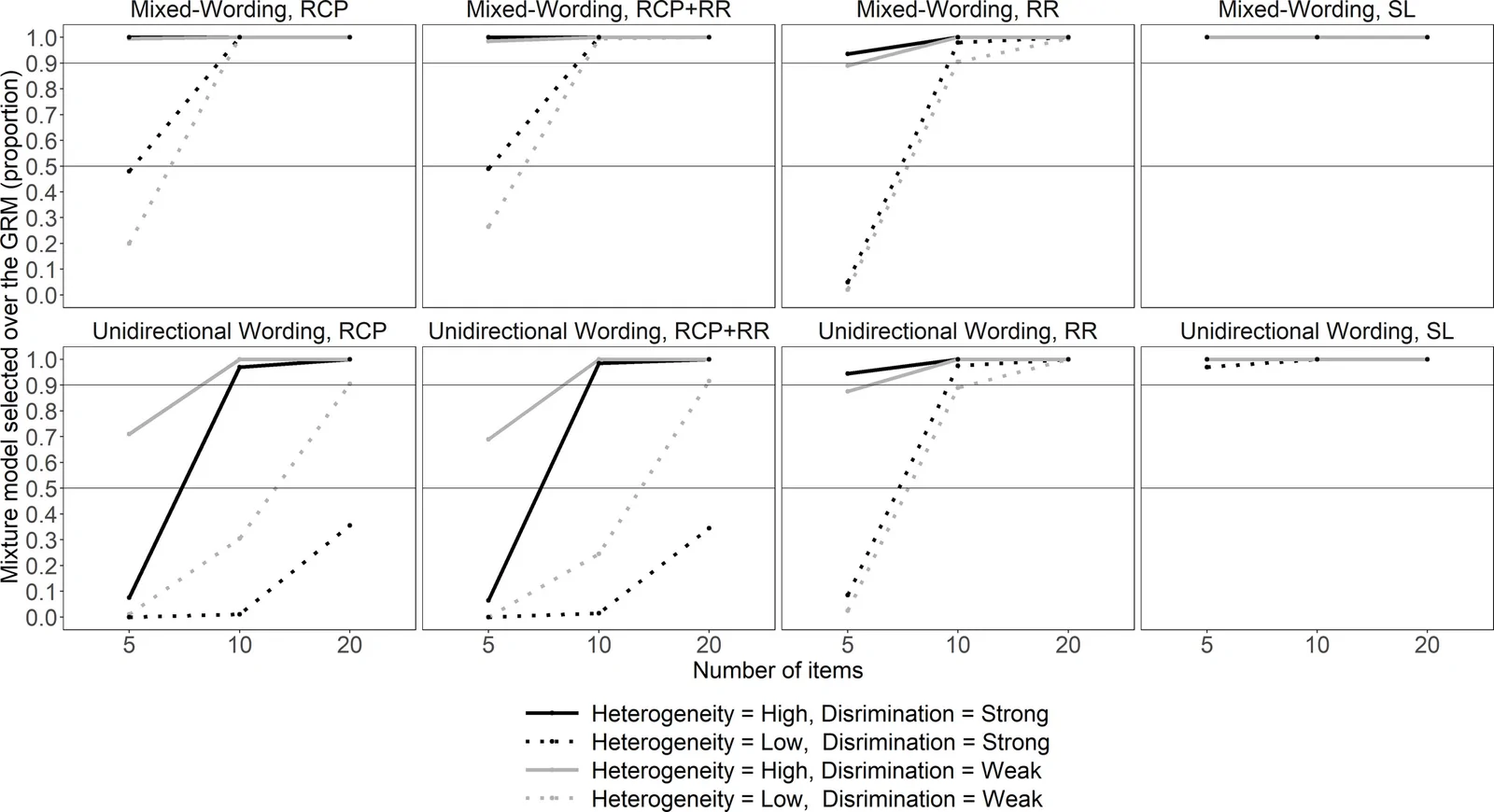
Proportion of cases where the mixture model was selected over the GRM. *Note*. The *horizontal lines* represent proportions of 0.50 and 0.90 of cases. *Solid lines* represent conditions where Heterogeneity = High; *black lines* represent conditions where Discrimination = Strong. RCP = respondent-specific category preference; RR = random responding; RCP + RR = respondent-specific category preference and random responding; SL = straightlining

Under all conditions with 0% C/IER, the BICs indicated the selection of the GRM over the mixture model, supporting the correct conclusion that the data were not affected by C/IER. Figure 2 shows the proportion of cases where the mixture model was selected when the data were contaminated with C/IER. Under SL conditions, the mixture model was consistently preferred in all conditions. In the vast majority of replications under RCP and RCP + RR, for shorter scales (five items), mixed-worded items with high item heterogeneity were required for consistent and correct decisions. For ten-item scales, trustworthy model selection was achieved either with mixed-worded items or with items with higher heterogeneity. For longer scales (20 items), the mixture model was preferred regardless of the items’ semantic polarity or the item heterogeneity, except for one condition (unidirectional item wording, low item heterogeneity, and strong discrimination). Under RR, the mixture model was selected if there were 10 or more items or if the item heterogeneity was high in all replications.

In summary, the mixture model did not falsely indicate the presence of C/IER if C/IER did not contaminate the data. If the data included 10% C/IER and the number of items was rather small (5 or 10), trustworthy model selection depended on the item heterogeneity and the items’ semantic polarity, as well as on the type of C/IER. As in practice the predominant response patterns under C/IER are generally unknown, researchers should at least consider around ten items that clearly differ in their marginal endorsement probabilities form each other (high item heterogeneity) and/or include positively and negatively worded formulations.

### Parameter recovery

**Fig. 3:**
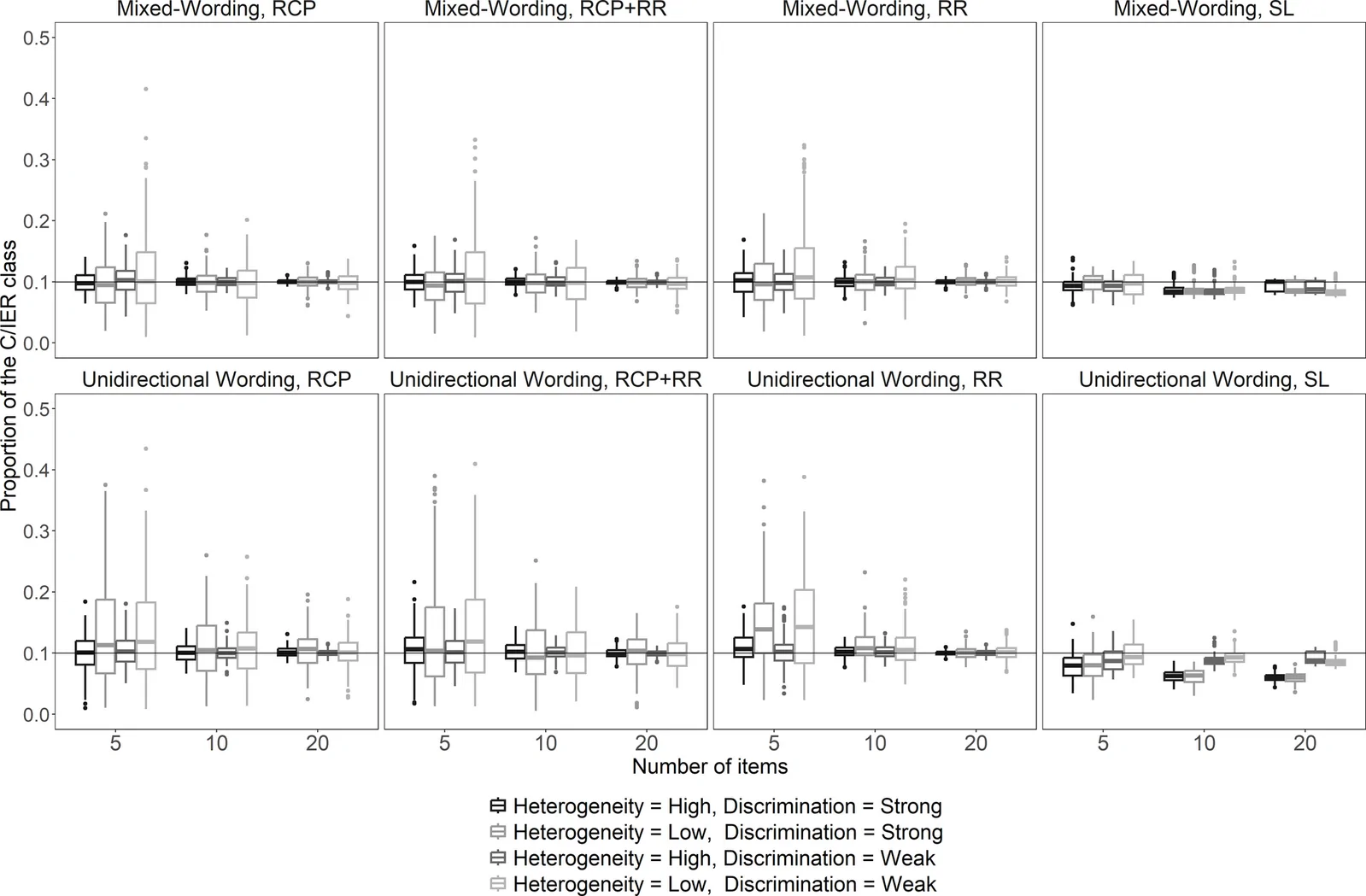
Estimated proportion of C/IER by condition. *Note*. The *horizontal line* indicates the true proportion of C/IER (0.1). RCP = respondent-specific category preference; RR = random responding; RCP + RR = respondent-specific category preference and random responding; SL = straightlining

*Proportion of C/IER.* The condition-specific estimated proportions of C/IER across replications are presented in Fig. 3. Under conditions where the C/IER class was represented by RCP, RR, or RCP + RR, the proportion of C/IER was within the range from 9 to 11%, if the items had mixed wording with increased variability for a shorter scale (five items). Under these C/IER patterns, the estimated proportion of C/IER was positively biased under conditions with unidirectionally worded items, low item heterogeneity, and shorter scales (five items), with the average estimated proportion of C/IER approaching 14%. Under the SL condition, the proportion of careless respondents was systematically underestimated, particularly for unidirectionally worded items with strong discrimination, with average estimates dropping as low as 6%. Notably, increasing the number of items did not improve the estimation of the C/IER proportion; on the contrary, the negative bias increased with scale length. This further highlights that straight-lining represents an extremely challenging condition for mixture model-based C/IER detection.

Because the C/IER patterns present in the data are unknown, scales that include both positively and negatively worded items are recommended. Alternatively, when only unidirectionally worded items are used, at least ten items with high item heterogeneity are recommended to ensure nonbiased estimation of the C/IER proportion.

Additionally, we inspected the parameter recovery of discrimination and threshold parameter estimates. The parameters were well recovered across the majority of conditions (see Supplementary Materials 2: https://osf.io/mzb3e/overview?view_only=4ce6d6aefcc042bea35bba96d9142522).

### Accuracy of individual-level classification

#### Specificity

Specificity values consistently exceeded .90 across all conditions. As specificity gives the probability of being assigned to the attentive class for the observations that were simulated as attentive, the model effectively assigned the majority of attentive respondents’ observations correctly to the dominant class.

#### Sensitivity

**Fig. 4:**
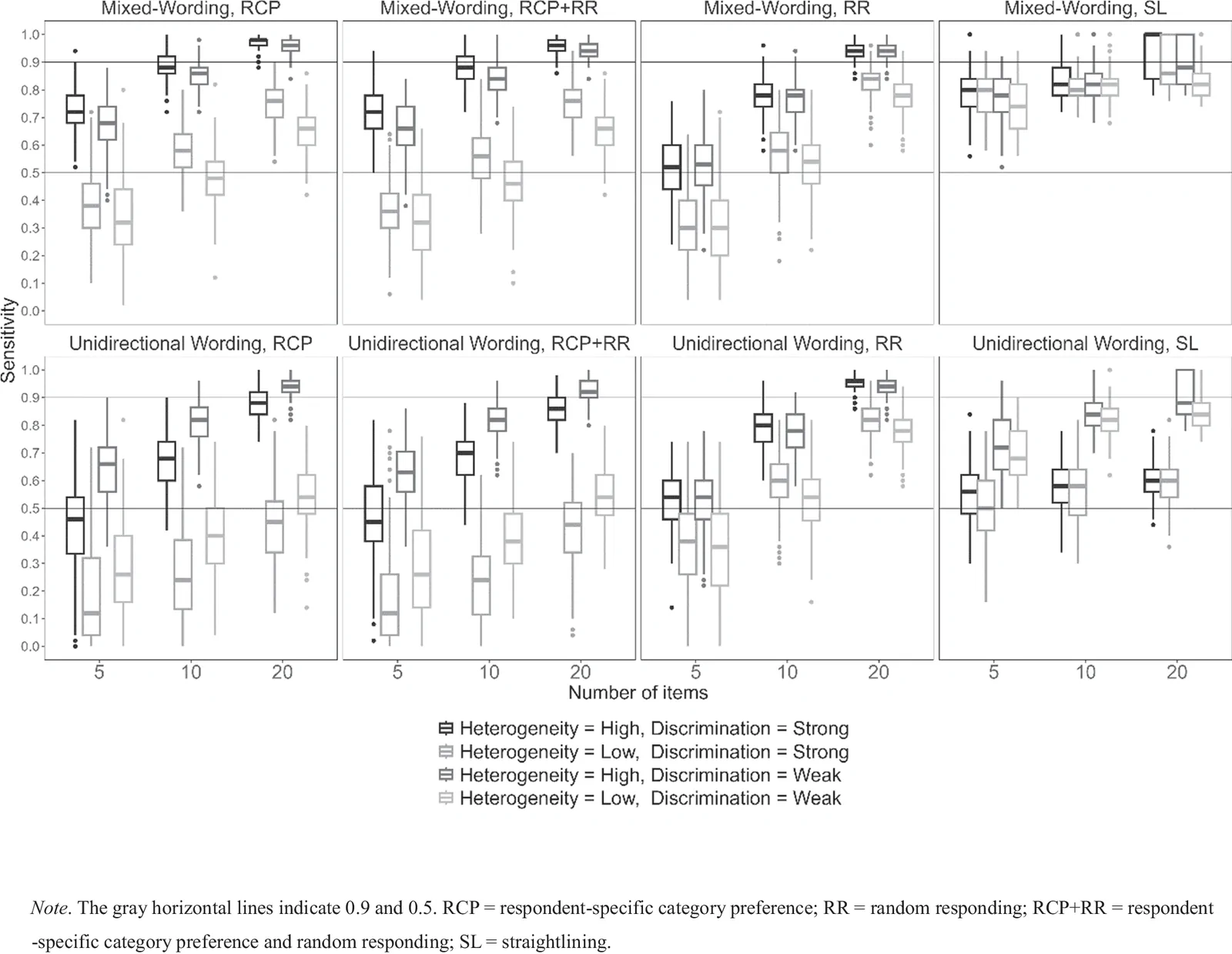
Sensitivity across conditions

Sensitivity indicates the model’s ability to assign true careless observations to the C/IER class. Figure 4 shows that sensitivity depended on the questionnaire characteristics and the type of C/IER. Under RR, RCP, and RCP+RR, sensitivity generally increased with scale length and item heterogeneity, and was higher for the mixed-wording condition. Under SL, sensitivity also increased with scale length, and the mixed-wording condition generally yielded higher sensitivity values than the unidirectional wording condition. However, for the unidirectional wording condition, discrimination played a more prominent role than item heterogeneity, with stronger discrimination slightly decreasing sensitivity. Because the type of C/IER is unknown in practice, scales with at least ten items, mixed wording, and high item heterogeneity are recommended. When only unidirectionally worded items are used, at least 20 items and high item heterogeneity generally provide more favorable conditions.

### Summary of the simulation study

In summary, a mixture model’s ability to separate careless and attentive response patterns depends primarily on three factors: questionnaire length, item heterogeneity, and the presence of items with different directions of wording. Particularly, conditions that support minimally biased estimation of the proportion of careless respondents include (a) scales with positively and negatively worded items or (b) for scales with unidirectionally worded items, at least ten items with high item heterogeneity. The condition that supports the correct classification of individual respondents is 20 high-heterogeneity items.

## Empirical application

By applying the exemplar mixture IRT model (Fig. 1, Eqs. 1, 2) to empirical data, we aimed to gather further validity arguments for the interpretation of the latent class variable to indicate response attentiveness. We reanalyzed a publicly available Big Five inventory data set, which includes attention check items collected from four online crowdsourcing platforms (Douglas et al., 2023). Corroborating previous research (Chmielewski & Kucker, 2020; Peer et al., 2022; Suri et al., 2025), Douglas et al. (2023) demonstrated that different data-collection platforms yielded different data quality, with Amazon Mechanical Turk (MTurk) showing the lowest data quality across various C/IER indicators. However, Douglas et al. (2023) derived their conclusion on the basis of combining different measures with indicators of C/IER.

In order to judge the validity of the interpretation of the latent class variable, first, we examined whether there was agreement between the classifications made by the exemplar mixture IRT model and by the attention check items. Second, we examined the consistency of the model’s classifications across scales to ensure the stability of observed behavior. Lastly, we evaluated whether model-based classifications showed a stronger association with administration platform than the attention check items.

### Data

The data published by Douglas et al. (2023) comprise responses across four^1^ platforms: MTurk (*n* = 500), CloudResearch (*n* = 505), Prolific (*n* = 496), and Qualtrics (*n* = 575). The sample consists of adult participants from the United States (for details, see Douglas et al., 2023, Table 1). The administered questionnaire is a 50-item version of NEO-PI-R traits (Costa & McCrae, 1992), measuring five traits. Each trait is measured by ten, five-point Likert scale items, with half of them being negatively worded. Based on these characteristics, the previous simulation study suggests that in the present application, our mixture model for C/IER (1) has a high power of detecting the presence of C/IER (Fig. 3), (2) is likely to provide solid estimates of the proportion of C/IER, and (3) offers a reasonable classification accuracy of individual C/IER (sensitivity).

Five attention check items were available in the dataset: (a) identifying a color mentioned in the consent form, (b) leaving a text entry box blank, (c) selecting “strongly agree” according to a direct instruction, (d) a colorblindness check (selecting “red-blue colorblindness”, which does not exist, indicated careless behavior), and (e) completing a simple arithmetic problem. We calculated the sum score of the attention-check items to create an attention-check index. All respondents passed at least one attention check item. A total of 0.9% passed two items, 8.4% passed three, 70.9% passed four, and 19.9% passed all attention check items. In addition, based on the empirical distribution, we computed a dichotomized attention check index and marked respondents who passed at least four items as attentive.

### Data analysis

First, we applied the mixture model described above separately to each NEO-PI-R scale, treating each scale as unidimensional. Estimating high-dimensional mixture models is computationally challenging and may lead to convergence issues within the *mirt* framework. The following analyses used the posterior probabilities of an individual’s membership in the attentive class, which were estimated separately for each scale. After that, we first examined the relationship between the posterior probabilities and the attention check index. Second, we examined the proportion of respondents who changed their class membership (respondents were assigned to the attentive class if their posterior probability exceeded 0.50). Additionally, Spearman correlation coefficients were calculated for posterior probabilities across scales to investigate the consistency of the classification. Lastly, to investigate whether the mixture model is more sensitive to C/IER than attention checks across different platforms, we compared Nagelkerke’s pseudo-${R}^{2}$ of two logistic regressions, which quantifies the strengths of relationships between data collection platforms (predictors) and (a) the model-based classifications and (b) the dichotomized attention check index, respectively (outcomes). Our focus was on the relative strength of the relationship between the classification approach and the data collection platform. Higher pseudo-*R*.^2^ values indicate stronger relationships with the platforms. The script for the model described above is available in the OSF repository: https://osf.io/mzb3e/?view_only=7c3575e6386c49e197c1c8b68918abb0

## Results

The proportion of respondents assigned to the attentive class by the mixture model varied across scales. Specifically, 76.5% were classified as attentive on the Extraversion scale, 77.9% on the Conscientiousness scale, 81.9% on the Agreeableness scale, 82.3% on the Neuroticism scale, and 83.3% on the Openness scale. We found that the mixture model consistently classified respondents across scales: A total of 62.4% of the respondents were classified as attentive across all five scales, and 8.4% were not classified as attentive on any scale; 15.7% were classified as attentive across four scales; 6.8% were classified as attentive on three scales; and only 3.4% and 3.3% were classified as attentive on two and one scale, respectively. The correlation coefficients between the posterior probabilities of being in the attentive class across scales were similar across different pairs of scales, varying from .509 (Agreeableness–Openness) to .666 (Conscientiousness–Neuroticism), with a mean of .566.

**Fig. 5:**
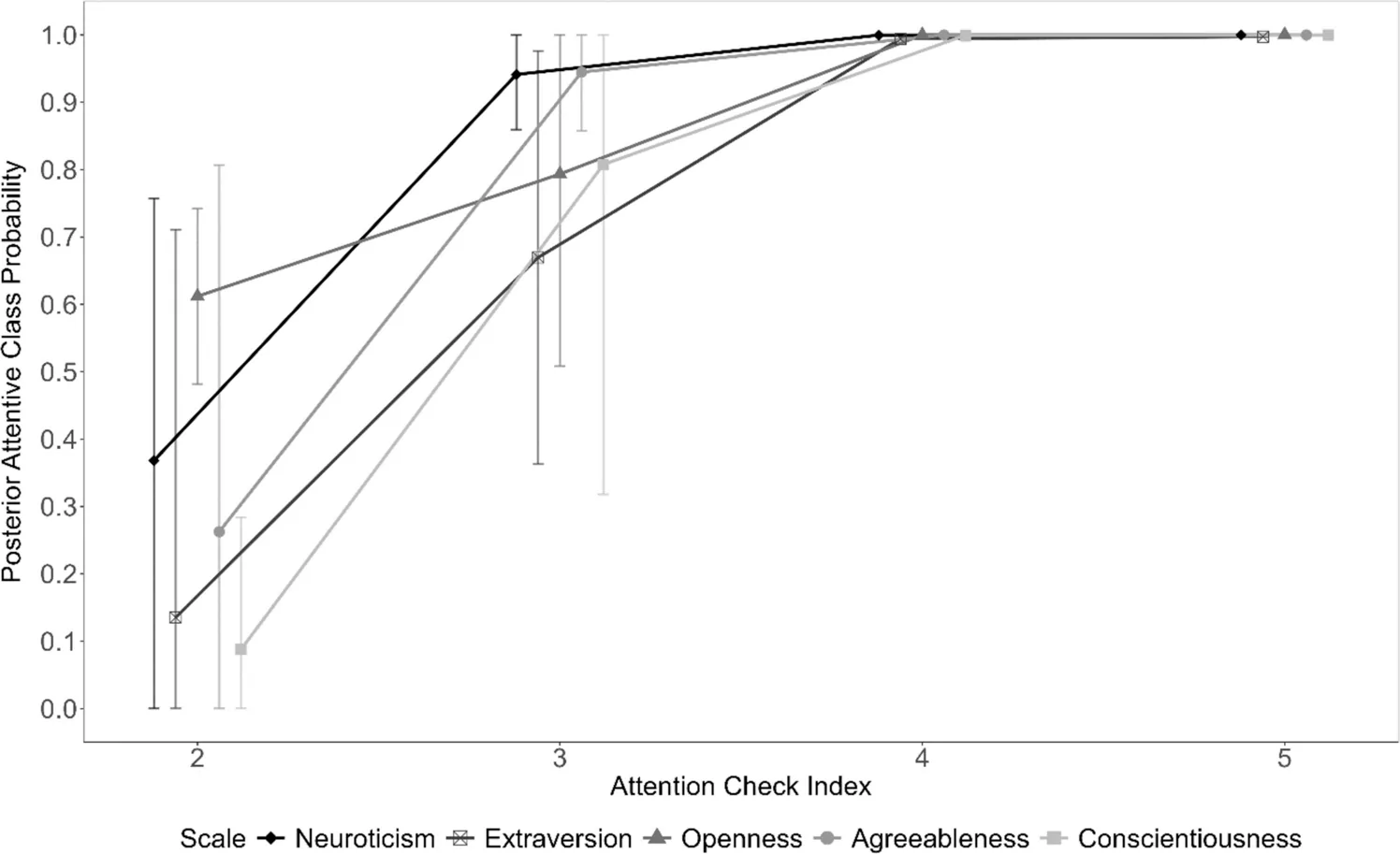
Posterior probability of being in the attentive class per scale, plotted against the Attention Check Index. *Note*. *Points* represent median posterior probabilities. *Error bars* represent the median absolute deviation (MAD). The Attention Check Index ranges from 2 to 5, reflecting the observed range in the sample (no respondents scored 0 or 1)

Figure 5 displays the median posterior probabilities of being in the attentive class for each scale across varying levels of the attention check index, along with median absolute deviation bars reflecting variability within each group. For respondents with attention check index scores of 2 and 3 (*n* = 19 and *n* = 174, respectively), posterior probabilities varied considerably across respondents, as reflected in the wide median absolute deviation (MAD) bars. For respondents who passed four or five attention check items (*n* = 1471 and *n* = 412, respectively), virtually all respondents across all scales received a median posterior probability of 1, indicating highly consistent classification as attentive. To formally examine whether the relationship between posterior probabilities and the attention check index differed across scales, we computed Spearman correlations for each scale and compared them against Openness as an exemplar baseline using dependent correlation tests accounting for the fact that all correlations were obtained from the same sample. All scales showed positive and statistically significant correlations with the attention check index: Openness (*r* =.196, 95% CI [.155, .237]), Neuroticism (*r* =.176, 95% CI [.134,. 218]), Extraversion (*r* =.156, 95% CI [.113, .197]), Conscientiousness (*r* =.155, 95% CI [.112, .196]), and Agreeableness (*r* =.114, 95% CI [.072, .156]). Tests indicated that the Openness correlation did not differ significantly from those of Neuroticism (*t* = 0.95, *p* =.34) or Extraversion (*t* = 1.96, *p* =.05), but was significantly higher than those of Conscientiousness (*t* = 1.98, *p* =.048) and Agreeableness (*t* = 3.84, *p* <.001).

**Fig. 6:**
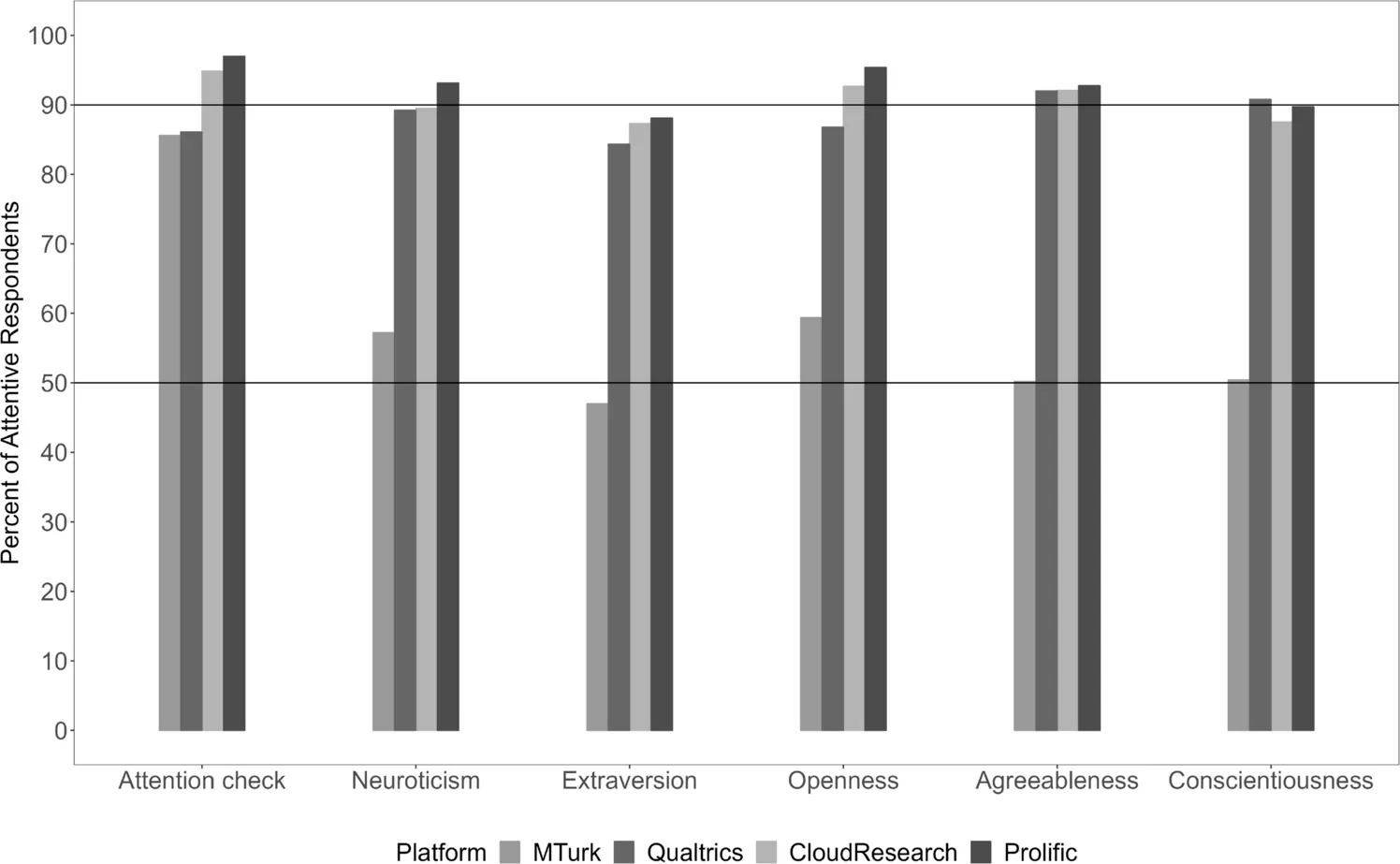
Percentage of attentive respondents by detection method and platform. *Note*. Attention check indicates the percentage of attentive respondents based on the dichotomized attention check index. Neuroticism, Extraversion, Openness, Agreeableness, and Conscientiousness indicate the percentage of attentive respondents assigned to the attentive class of the mixture model. The *horizontal lines* indicate 50% and 90% of attentive respondents, respectively

To visualize the strength of the relationship between C/IER classification and the data collection platform, Fig. 6 shows the percentage of respondents assigned to the attentive class by the mixture model and marked as attentive by the attention check index. The proportions varied across platforms and differed between the attention check index and the posterior probabilities. Results based on posterior probabilities were replicated across scales, consistently indicating that MTurk yielded, by a large margin, the lowest rate of attentive respondents compared to the other platform. Based on the attention check index, the proportion of attentive respondents varied from 85.6% (MTurk) to 96.9% (Prolific), while, based on the posterior probabilities, the proportion varied from 47.0% (MTurk) to 88.1% (Prolific) on the Extraversion scale and from 59.4% (MTurk) to 95.4% (Prolific) on the Openness scale. The results of the logistic regression analysis showed that the relationship between the survey administration platforms and the mixture model’s classification was stronger compared to the classification based on the attention check index. Nagelkerke’s pseudo-${R}^{2}$ value, averaged across scales, was 0.224 for the model-based classification and 0.071 for the index-based classification. That is, the model-based classification showed a stronger association with data-collection platform than the Attention Check Index.

To summarize, the interpretation of the latent class variable as a measure of C/IER class was supported by the association between the model-based classification and the responses to the attention check items. Moreover, the mixture-model classifications were highly consistent across scales. Respondents who were identified as careless on one scale were also likely to be identified as careless on the other scales, indicating stable careless responding rather than scale-specific anomalies. Finally, the model-based classification showed a stronger association with the data collection platform than the classification based on attention check items.

## Discussion

The overarching goal of this article is to advance the understanding of the potential and limitations of two-class mixture models for C/IER in applied research. Drawing on a large simulation study, we examined conditions that facilitate or impede the separability and identification of response patterns commonly considered indicative of C/IER. In addition, by reanalyzing existing data, we contributed to the growing body of research examining the validity of interpreting the latent class variable in terms of C/IER. We conducted all analyses using a modified two-class mixture model that avoids several debatable assumptions of existing approaches, including treating categorical item responses as continuous in linear measurement models and making strong assumptions about dependencies among nonattentive responses. Accordingly, the present findings are expected to generalize to closely related mixture models. Moreover, the employed model constitutes a practically useful and comparatively easy-to-implement approach for addressing C/IER in a wide range of applied settings.

The simulation study covered a broad range of conditions that can facilitate or impede the identification of C/IER. A central focus was the role of prevailing response patterns commonly considered characteristic of C/IER. This focus is critical because, as the results show, response patterns exert a relatively strong influence on model performance. In applied settings, however, the dominant form of C/IER is typically unknown. Consequently, the present findings help identify conditions under which mixture models are most likely to perform adequately even in the presence of challenging response patterns.

Whereas the simulation study identified conditions under which latent groups can be separated based on their response patterns, the empirical application contributed to the growing body of evidence regarding the substantive interpretation of the latent class variable as reflecting C/IER. The robustness of the findings is supported by the fact that the analyzed scales met the requirements identified in the simulation study, namely the use of at least 10 items with both positively and negatively worded statements and sufficient item heterogeneity. Across scales, individual-level classifications showed a high degree of consistency, indicating that the identified latent class membership did not reflect scale-specific idiosyncrasies. Moreover, latent class membership was strongly associated with alternative indicators of C/IER that do not rely on item response patterns, thereby reducing the plausibility of common method bias as an explanation for the observed associations. The results were consistent across scales, with a notable exception of a significant difference between Openness, which yielded the highest correlation, and Agreeableness, which yielded the lowest. As the item parameters of the scales are comparable in terms of both discrimination and threshold estimates (see Appendix C), unusual psychometric properties do not appear to account for the differences in correlations observed across scales. Other factors, such as model misspecification, may have contributed to differences among scales (Vogelsmeier et al., 2024), though with empirical data it is inherently impossible to determine whether observed differences reflect misspecification or true variation in C/IER behavior, as the ground truth is unknown.

In addition, class membership exhibited a pronounced relationship with the data collection platform. Notably, this association was stronger than the corresponding relationship observed for attention checks, suggesting that model-based latent class scores provide a more precise and sensitive representation of C/IER. Taken together, the empirical findings not only strengthen trust in the validity of interpreting the latent class variable in terms of C/IER but also illustrate the practical utility of the proposed modeling approach. Specifically, the results demonstrate that applied researchers can benefit from an effective and comparatively easy-to-use latent variable framework for addressing C/IER in self-report data.

### Practical recommendations

Methodological research has consistently emphasized that the potential presence of C/IER should not be ignored (Meade & Craig, 2012; Nichols & Edlund, 2020; Ward & Meade, 2023). Although it is still the case that researchers have limited options to prevent C/IER (Gibson & Bowling, 2020; Ulitzsch et al., 2024a, 2024b; Ward & Meade, 2018), our simulation results demonstrate that it is possible to create conditions that ensure the optimal functioning of response-pattern-based C/IER detection tools. To create such conditions, we recommend using longer scales (ten or more items), including mixed-worded items, and, whenever possible, incorporating items with varying degrees of response threshold differences to increase item heterogeneity. If researchers do not want to include negatively worded items in their questionnaires (see Arias et al., 2023; Dalal & Carter, 2014, for discussions on potential drawbacks), we recommend focusing on scales with greater item heterogeneity, meaning scales that cover a broader range of the underlying trait and can more effectively differentiate among individuals (De Boeck, 2008; Reise et al., 2005). This requirement can be expected to be fulfilled in many widely used questionnaires, such as the Big Five Inventory used in the empirical part of this study.

It is important to note that the present study does not examine the ability of two-class mixture models for C/IER to accurately estimate individual-level latent trait scores in the presence of C/IER. Rather, its primary contribution lies in identifying the questionnaire and item characteristics that facilitate or hinder latent class separation, thereby illuminating the conditions under which an interpretation of the latent class variable as C/IER is more strongly supported, as well as gathering further empirical evidence for the validity of interpreting the latent class variable in terms of C/IER. As illustrated in the simulation study, the evaluated mixture model provided useful tools to determine the extent to which data are contaminated by C/IER under many conditions encountered in practice. Furthermore, as demonstrated by the empirical application, under such conditions, the mixture models are likely to identify C/IER rather than response patterns resulting from other processes.

As the mixture model examined effectively separates attentive from non-attentive respondents, it improves trait estimates in the sense of identifying respondents whose data do not allow their trait levels to be reliably determined. However, the extent to which two-class mixture models improve trait estimates relative to conventional measurement models likely hinges on various factors and their interactions (e.g., C/IER prevalence, individual-level classification accuracy, etc.), which lie beyond the scope of this paper., This issue clearly warrants further attention in future studies.

The simulation study and the empirical application demonstrated the usefulness of mixture modeling for C/IER detection. Moreover, mixture modeling avoids reliance on researcher-chosen classification thresholds; instead, it quantifies classification uncertainty via posterior class probabilities.

Applied researchers should also be aware that under unfavorable conditions, such as short scales, all items having the same direction of wording, and low threshold heterogeneity, the model’s classification accuracy is reduced, as reflected primarily in lower sensitivity. This is partly a consequence of class imbalance: with only 10% C/IER respondents in the data, a classifier that assigns all respondents to the attentive class would already achieve 90% specificity, meaning that reduced accuracy is much more visible in sensitivity than in specificity. Under such conditions, misclassifications occur in both directions—some C/IER respondents are incorrectly classified as attentive, and some attentive respondents may be incorrectly classified as C/IER. The practical consequences of either type of misclassification remain to be determined in future research. This is a particularly pertinent question because misclassified respondents are unlikely to constitute a random subsample; for instance, respondents with extreme trait levels may be more likely to be misclassified as straight-liners.

A further practical advantage is that the class of mixture models examined in this article is straightforward to implement in standard statistical software (e.g., the *mirt* package in R). The script for the models described above is available in the OSF repository: https://osf.io/mzb3e/?view_only=7c3575e6386c49e197c1c8b68918abb0 and can easily be adapted to other IRT-based mixture modeling approaches by modifying constraints of the C/IER class. For practical usage, we note that as the variance of the latent variable in the C/IER class is expected to be very large under SL conditions, we recommend estimating the model using both the default and very large starting values for the variance of the latent variable in the C/IER class (e.g., 1000). The solution with the largest LL should then be selected.

### Limitations and future directions

The aim of the present study was to systematically evaluate conditions that facilitate C/IER detection within mixture-modeling frameworks. The model used here integrates key features of previously proposed mixture models (Arias et al., 2020; Arias et al., 2023; Kam & Cheung, 2023; Steinmann et al., 2022; van Laar & Braeken, 2022; Ulitzsch et al., 2022a, 2022b). However, a direct empirical comparison of different mixture-model specifications across a broad range of conditions remains an important task for future research.

The agreement between the classification of attention check items and the model’s classification in the empirical application supports the interpretation of the latent class variable in terms of C/IER. However, although the latent class variable showed stronger associations with the survey platforms than the attention check indices, this finding does not provide direct evidence as to which indicator constitutes the more effective measure of C/IER. To gain further insight into the relative effectiveness of different C/IER indicators, study designs offering unambiguous validity criteria are warranted. These could be experimental studies using varied instructions, for example, warning messages aimed at reducing C/IER (Bowling et al., 2021) or instructions prompting some participants to complete the questionnaire as quickly as possible without reading the items (Roman et al., 2024), or studies manipulating mental fatigue (e.g., Jeong et al., 2023; Meier et al., 2024), which directly affect the prevalence of C/IER.

Mixture models for C/IER detection depend on strong assumptions, such as the correct specification of the attentive measurement model. For instance, if the dimensionality of the attentive class is misspecified, the estimated C/IER proportion may be biased (Vogelsmeier et al., 2024). Moreover, the simulation and empirical studies were focused on unidimensional cases. Extending the investigation to multidimensional structures remains an important direction for future research.

Furthermore, there are specific situations in which response patterns such as long strings of extreme scores on a scale containing only positively worded items can plausibly be attributed to either attentive or careless responding, making the latent classification ambiguous. Incorporating additional information that reflects differences in respondents’ behavior, such as response times (Bowling et al., 2023; Ulitzsch et al., 2022a, 2022b, 2024a, 2024b), mouse movements (Pokropek et al., 2023), or item position (Ulitzsch et al., 2022a, 2022b), may enhance C/IER identification even when these assumptions are not fully met. The investigation of the potential of including such auxiliary information (e.g., as predictors of the latent class) to facilitate C/IER identification is a pertinent topic for future simulation studies.

Because the mixture model described above and related mixture models for C/IER (e.g., Arias et al., 2023; Kam & Cheung, 2023) absorb a variety of C/IER patterns into a single careless class, the parameters of the careless class are not interpretable, as they reflect a mixture of multiple C/IER response patterns. If researchers are interested in investigating the specific types of C/IER displayed, other approaches may be more appropriate. The approach provided by Jin et al. (2018) and Jin and Chiu (2024), for instance, assumes that careless respondents can be divided into different classes that represent different types of C/IER, assumed a priori, and, therefore, allows researchers to investigate the presence of these different types of C/IER patterns. Moreover, in the present article we focused on individual-level output in terms of posterior class probabilities. Evaluating the accuracy of estimating where a respondent lies on the substantive latent construct when accounting for C/IER is an important direction for future research.

The mixture models considered in the present article identify C/IER at the respondent level. However, previous research has repeatedly shown that respondents’ behavior may vary within a questionnaire. This trend has been documented by self-reports (Baer et al., 1997; Berry et al., 1992), infrequency items (Bowling et al., 2021, 2023), and mixture modeling (Roman et al., 2024; Ulitzsch et al., 2022a, 2022b, 2024a, 2024b). Mixture models can be extended to identify C/IER at the respondent-by-item level by adding a dynamic component (Roman et al., 2024; Ulitzsch et al., 2022a, 2022b); however, this makes the model more complicated to apply. Therefore, such dynamic modeling may not always be desirable. Moreover, the decision in favor of C/IER identification at a respondent-by-item level or at a respondent level depends on the aim of the analysis. On the one hand, respondent-by-item identification provides finer-grained insights into how C/IER occurrence evolves across the questionnaire and makes it possible to identify item characteristics such as text length that are associated with a higher risk of C/IER occurrence (e.g., Ulitzsch et al., 2022a, 2022b). On the other hand, if researchers are more interested in evaluating whether or not a respondent’s response patterns can be trusted, respondent-level identification may be more appropriate.

In this article, we focused on careless respondents as a prominent source of aberrant responses that bias results. Another source of increasing concern is bots (also known as automated form fillers), which are computer programs that automatically complete web-based surveys (Irish & Saba, 2023; Xu et al., 2022). It has been shown that mixture models can also be a promising tool for bot detection (Roman et al., 2022). Further explorations of the potential of mixture models for this purpose pose an interesting topic for future research.

## Supplementary Information

Below is the link to the electronic supplementary material.

Supplementary file1 (DOCX 525 kb)

## Data Availability

The study is based on secondary analysis of publicly available data from Douglas, B. D., Ewell, P. J., & Brauer, M. (2023). Data quality in online human-subjects research: Comparisons between MTurk, Prolific, CloudResearch, Qualtrics, and SONA. *PLOS ONE, 18*(3), e0279720. The data used in this study are publicly available at: https://osf.io/ern4u/ (https://osf.io/z2s9a).

## Footnotes

1 Originally, the study included one more online platform, SONA. The participant pool on SONA included students who participated for course credit. The data collected from SONA is not publicly available.

## Code availability

Analyses were conducted using the “*mirt*” package in R. The corresponding script is available in the OSF repository: https://osf.io/mzb3e/?view_only=7c3575e6386c49e197c1c8b68918abb0.
