# Supplementary material for: Understanding when mixture models effectively identify careless responding: Simulation and validity evidence
Source: Behav Res Methods. 2026 Oct 9;58(11):313. doi: 10.3758/s13428-026-03165-z (PMC13652882; doi:10.3758/s13428-026-03165-z)
Supplement: Supplementary file 1 [file 13428_2026_3165_MOESM1_ESM.docx]

**Appendix A**

Appendix A elaborates on how the mixture model accommodates different types of C/IER within its “catch-all careless” class. Specifically, we describe how the model absorbs atypical response patterns arising from straightlining (SL), respondent-specific category preferences (RCP), and random responding (RR).

**Straightlining**

SL means that respondents choose the same response category throughout the considered scales. Figure A1 illustrates how SL is approximated by the mixture model. As demonstrated, the probability of a respondent choosing category *k* essentially differs from 0 only within a specific relevant interval of the horizontal axis $\zeta$. The thresholds $\beta_{01}$, …, $\beta_{0K-1}$, which are represented by the vertical lines in Figure A1, are spaced very far apart. The thresholds indicate the levels of the latent variable$\zeta$ at which a respondent has a 50% probability of selecting category *k* or lower categories. Additionally, the variance of $\zeta$ is expected to be very large.

**Figure A1** Representation of item characteristic curves for straightlining

*Note*. *Vertical lines* indicate the thresholds $\beta_{01}$, …, $\beta_{0K-1}$.

**Respondent-specific category preference**

In this pattern, the probability of selecting a specific category is high, but the probability of selecting other categories also remains above zero. SL can be seen as an extreme form of category preference, where the latent category preference entirely determines the observed responses. As illustrated by the item characteristic curves (ICCs) in Figure A2, the thresholds $\beta_{01}$, …, $\beta_{0K-1}$, indicated by vertical lines, are spaced far apart but a respondent can choose a category *k* or a category lower than *k* with a high probability. In the given example, categories “3”, “4”, and especially “5” are most preferred by the majority of respondents. This preference is not influenced by the specific content of the items but is instead determined by the latent category preference variable $\zeta$.

**Figure A2** Representation of item characteristic curves for respondent-specific category preference

*Note*. *Vertical lines* indicate the thresholds $\beta_{01}$, …, $\beta_{0K-1}$.

**Random responding**

If respondents do not differ in their category preferences, careless responses are driven by category probabilities that generalize across all respondents. Hence, RR is accommodated by the model when the variance of $\zeta$ is estimated to be close to zero. In this case, Eq. (2) will essentially reduce to

$P\left( x_{ij}\geq k | C_{i}=0 \right)=1\text{/}\left[ 1+exp\left( -\left( \beta_{0k} \right) \right) \right]$	(A1).

Noticeably, Equation A1 resembles previous mixture IRT models (van Laar & Braeken, 2022; Ulitzsch, Yildirim-Erbasli, et al., 2022) except that the model by van Laar and Braeken (2022) constrains response options to have an equal probability of being chosen. Therefore, the mixture model applied in the present article incorporates the careless component of previous mixture IRT models (van Laar & Braeken, 2022; Ulitzsch, Yildirim-Erbasli, et al., 2022) as special cases.

**Appendix B**

**Figure B1** Item characteristic curves for item 1 and item 5 in different simulation conditions

*Note.* The black vertical line indicates 0, the gray vertical lines indicate -1.5 and 1.5.

**Appendix C**

**Table C1** Item parameters of the NEO-PI-R scales

| Item | *α*_1j_ | *β*_1j1_ | *β*_1j2_ | *β*_1j3_ | *β*_1j4_ |
| --- | --- | --- | --- | --- | --- |
| Neuroticism | | | | | |
| n_1 | – 5.052 | 1.979 | – 1.605 | – 3.928 | – 8.186 |
| n_2 | – 3.166 | 0.255 | – 2.187 | – 3.850 | – 6.508 |
| n_3 | – 5.535 | 1.839 | – 2.336 | – 4.838 | – 9.628 |
| n_4 | – 2.366 | 1.019 | – 1.107 | – 2.558 | – 5.497 |
| n_5 | – 2.013 | 1.139 | – 0.757 | – 1.992 | – 4.208 |
| n_6r | 1.406 | 2.944 | 0.689 | – 0.388 | – 2.779 |
| n_7r | 2.127 | 3.108 | 1.146 | – 0.053 | – 2.374 |
| n_8r | 2.621 | 5.985 | 3.652 | 1.927 | – 1.585 |
| n_9r | 1.517 | 3.233 | 0.934 | – 0.287 | – 2.603 |
| n_10r | 1.957 | 4.162 | 2.139 | 0.067 | – 2.753 |
| Extraversion | | | | | |
| e_1 | – 2.645 | 4.918 | 2.377 | 0.631 | – 2.619 |
| e_2 | – 2.494 | 4.179 | 1.847 | 0.027 | – 3.090 |
| e_3 | – 2.594 | 4.467 | 2.110 | 0.003 | – 3.362 |
| e_4 | – 1.899 | 1.315 | – 0.619 | – 2.612 | – 5.106 |
| e_5 | – 2.129 | 3.314 | 1.068 | – 1.280 | – 4.300 |
| e_6r | 2.181 | 2.443 | – 0.003 | – 1.647 | – 4.303 |
| e_7r | 2.451 | 4.069 | 1.914 | 0.059 | – 2.983 |
| e_8r | 1.441 | 1.642 | – 0.338 | – 1.572 | – 4.087 |
| e_9r | 1.409 | 4.132 | 2.395 | 0.900 | – 1.183 |
| e_10r | 2.400 | 3.242 | 0.956 | – 0.673 | – 3.305 |
| Openness | | | | | |
| o_1 | 2.806 | 5.649 | 4.195 | 2.312 | – 0.927 |
| o_2 | 1.059 | 3.686 | 2.060 | 1.035 | – 0.969 |
| o_3 | 0.826 | 1.231 | 0.684 | – 0.080 | – 0.965 |
| o_4 | 0.810 | 2.819 | 1.528 | 0.050 | – 2.080 |
| o_5 | 1.323 | 5.338 | 3.739 | 2.130 | – 0.759 |
| o_6r | – 1.539 | 1.142 | – 0.685 | – 1.880 | – 3.582 |
| o_7r | – 3.374 | – 0.255 | – 3.144 | – 4.591 | – 6.702 |
| o_8r | – 1.192 | 1.276 | – 0.409 | – 1.365 | – 2.850 |
| o_9r | – 2.298 | 0.705 | – 1.403 | – 2.420 | – 3.860 |
| o_10r | – 0.745 | 0.457 | – 0.066 | – 0.774 | – 1.711 |
| Agreeableness | | | | | |
| a_1 | 1.644 | 4.460 | 2.425 | 0.618 | – 2.455 |
| a_2 | 1.661 | 4.271 | 2.450 | 0.862 | – 2.539 |
| a_3 | 1.690 | 6.180 | 4.875 | 3.561 | 0.116 |
| a_4 | 1.457 | 5.320 | 3.699 | 2.169 | – 1.023 |
| a_5 | 1.187 | 4.128 | 2.790 | 1.321 | – 1.336 |
| a_6r | – 1.134 | 1.090 | – 0.406 | – 1.426 | – 3.346 |
| a_7r | – 1.976 | – 1.212 | – 3.371 | – 4.442 | – 6.253 |
| a_8r | – 1.263 | 1.998 | 0.195 | – 0.970 | – 3.185 |
| a_9r | – 1.370 | 0.255 | – 1.537 | – 2.517 | – 4.160 |
| a_10r | – 2.311 | – 0.767 | – 3.224 | – 4.551 | – 7.659 |
| Conscientiousness | | | | | |
| c_1 | 1.643 | 5.128 | 2.723 | 1.150 | – 2.000 |
| c_2 | 1.067 | 5.704 | 3.660 | 2.459 | – 0.319 |
| c_3 | 1.623 | 3.801 | 1.574 | 0.619 | – 1.796 |
| c_4 | 2.534 | 7.056 | 4.566 | 3.011 | – 1.364 |
| c_5 | 2.457 | 6.639 | 4.127 | 2.477 | – 1.450 |
| c_6r | – 1.903 | 1.000 | – 0.942 | – 2.018 | – 4.597 |
| c_7r | – 2.490 | 1.249 | – 1.642 | – 2.848 | – 5.686 |
| c_8r | – 1.399 | 0.676 | – 1.161 | – 2.196 | – 4.027 |
| c_9r | – 3.161 | 0.626 | – 3.046 | – 4.784 | – 8.440 |
| c_10r | – 1.753 | 0.033 | – 2.181 | – 3.534 | – 5.840 |

*Note***.** *α*_1j_ = discrimination parameter; *β*_1jk_ = threshold parameters (*k* = 1–4). Items marked with ‘r’ are reverse-coded.
